# Comorbidities and laboratory parameters associated with SARS-CoV-2 infection severity in patients from the southeast of Mexico: a cross-sectional study

**DOI:** 10.12688/f1000research.74023.2

**Published:** 2022-04-27

**Authors:** Eduardo De la Cruz-Cano, Cristina del C Jiménez–González, José A Díaz-Gandarilla, Carlos J López–Victorio, Adelma Escobar-Ramírez, Sheila A Uribe-López, Elizabeth Huerta-García, Jorge-Tonatiuh Ayala-Sumuano, Vicente Morales-García, Liliana Gútierrez-López, José A González-Garrido

**Affiliations:** 1 División Académica de Ciencias Básicas. CICTAT. Laboratorio de Bioquímica y Biología Molecular., Universidad Juárez Autónoma de Tabasco, Cunduacán,, Tabasco., 86690, Mexico; 2 Laboratorio de Análisis Clínicos., Secretaría de Salud, Hospital General de Comalcalco., Comalcalco., Tabasco, 86300, Mexico; 3 División Académica Multidisciplinaria de Comalcalco. Laboratorio de Análisis Clínicos., Universidad Juárez Autónoma de Tabasco., Comalcalco., Tabasco., 86650, Mexico; 4 División Académica Multidisciplinaria de Jalpa de Méndez. Laboratorio de Inmunología y Microbiología Molecular., Universidad Juárez Autónoma de Tabasco, Jalpa de Méndez, Tabasco, 86205, Mexico; 5 IDIX Biotechnology., IDIX Biotechnology, SA. de C.V, Querétaro, Querétaro, 76235, Mexico; 6 Sección de Estudios de Posgrado e Investigación, Escuela Superior de Medicina., Instituto Politécnico Nacional., Ciudad de México, Ciudad de México, 11340, Mexico

**Keywords:** SARS-CoV-2; type 2 diabetes mellitus; hypertension; obesity; laboratory parameters.

## Abstract

**Background** **. **Severe acute respiratory syndrome-coronavirus 2 (SARS-CoV-2) is the etiological agent of the coronavirus disease 2019 (COVID-19) pandemic. Among the risk factors associated with the severity of this disease is the presence of several metabolic disorders. For this reason, the aim of this research was to identify the comorbidities and laboratory parameters among COVID-19 patients admitted to the intensive care unit (ICU), comparing the patients who required invasive mechanical ventilation (IMV) with those who did not require IMV, in order to determine the clinical characteristics associated with the COVID-19 severity.

**Methods. **We carried out a cross-sectional study among 152 patients who were admitted to the ICU from April 1 ^st^ to July 31 ^st^, 2021, in whom the comorbidities and laboratory parameters associated with the SARS-CoV-2 infection severity were identified. The data of these patients was grouped into two main groups: “patients who required IMV” and “patients who did not require IMV”. The nonparametric Mann–Whitney U test for continuous data and the *χ* *2 *test for categorical data were used to compare the variables between both groups.

**Results. **Of the 152 COVID-19 patients who were admitted to the ICU, 66 required IMV and 86 did not require IMV. Regarding the comorbidities found in these patients, a higher prevalence of type 2 diabetes mellitus (T2DM), hypertension and obesity was observed among patients who required IMV vs. those who did not require IMV ( *p<0.05*). Concerning laboratory parameters, only glucose, Interleukin 6 (IL-6), lactate dehydrogenase (LDH) and C-reactive protein (CRP) were significantly higher among patients who required IMV than in those who did not require IMV ( *p<0.05*).

**Conclusion. **This study performed in a Mexican population indicates that comorbidities such as: T2DM, hypertension and obesity, as well as elevated levels of glucose, IL-6, LDH and CRP are associated with the COVID-19 severity.

## Abbreviations

ACE-2: angiotensin-converting enzyme 2

ALT: alanine transaminase

AST: aspartate transaminase

COPD: chronic obstructive pulmonary disease

COVID-19: coronavirus disease-2019

CKD: chronic kidney disease

CRP: C-reactive protein

EWS: Early Warning Score

FiO _2_: fraction of inspired oxygen

ICU: intensive care unit

IL-1β: Interleukin 1 beta

IL-6: interleukin 6

IMV: invasive mechanical ventilation

MSQ: Medical Symptom Questionnaire

PaO _2_: partial pressure of oxygen

PCR: polymerase chain reaction

RAAS: Renin-Angiotensin-Aldosterone System

SARS-CoV-2: severe acute respiratory syndrome-coronavirus 2

SCQ: Self-administered Comorbidity Questionnaire

spO _2_: blood oxygen saturation

T2DM: type 2 diabetes mellitus

TNFα: *tumor necrosis factor alpha*

WHO: World Health Organization.

## 1. Introduction

**Figure 1.**
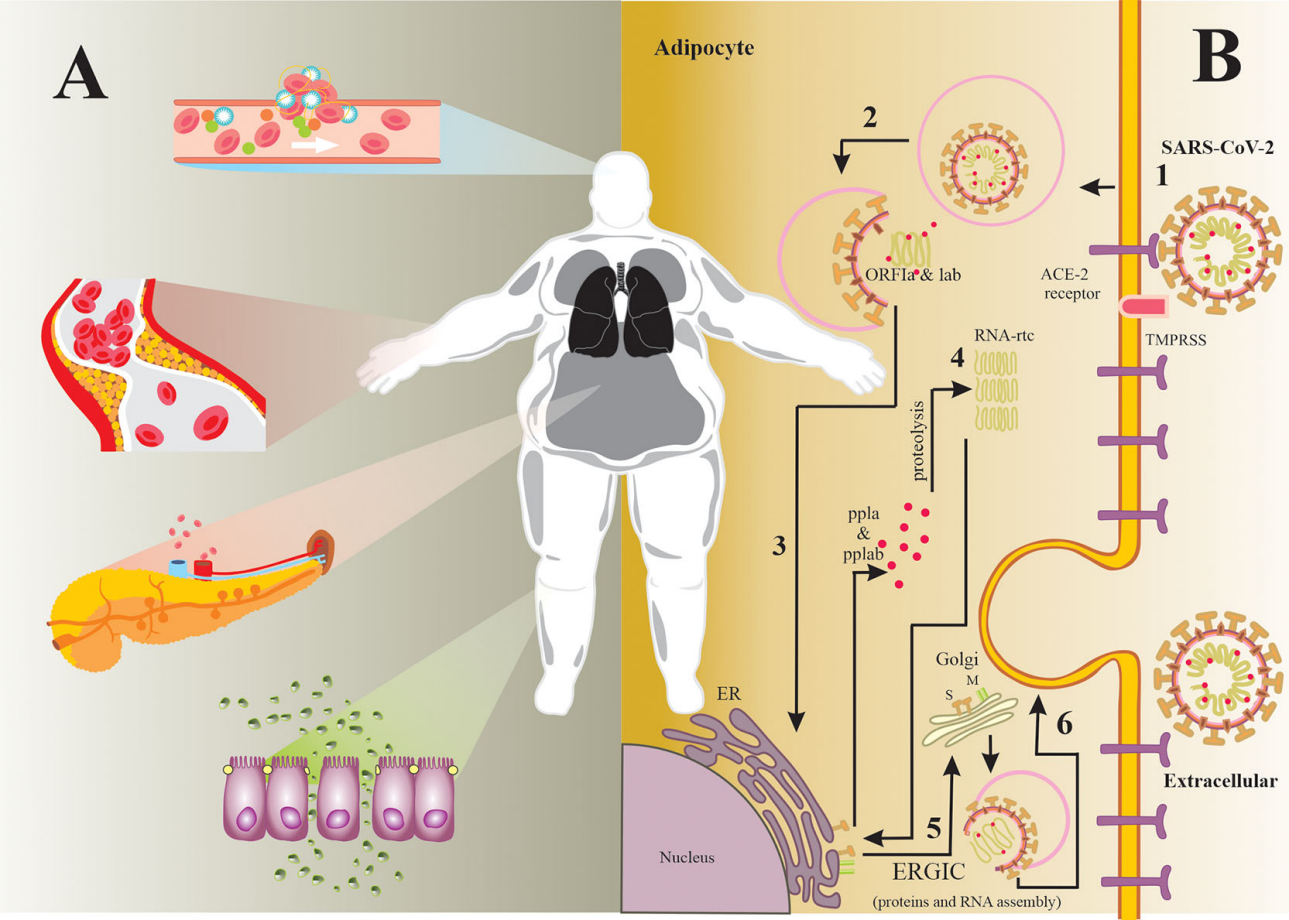
*(A) Pathophysiological characteristics that contribute to the COVID-19 severity.* It is well known that obesity is the major risk factor for other conditions such as: T2DM, hypertension and cardiovascular diseases. These entities share common characteristics that contribute substantially to the COVID-19 severity, For instance: endothelial dysfunction, coagulation system alterations, insulin resistance, increased pro-inflammatory mediators, among others. *(B) SARS-CoV-2 infection mechanism in the adipocyte.* 1) The SARS-CoV-2 S glycoprotein binds more easily to ACE-2 receptor in the adipocyte surface, since the expression levels of this receptor are higher in adipose tissue than in alveolar cells. Then, TMPRSS2 activates S glycoprotein by proteolysis to promote membrane fusion. 2) SARS-CoV-2 enters the adipocyte by endocytosis and releases its RNA genome in the cytoplasm. 3) ORF1a and ORF1ab are translated into polyproteins by the cell machinery. 4) These polyproteins are cleaved via proteolysis to produce structural proteins by the RNA replicase-transcriptase complex. 5) These structural proteins and viral RNA are assembled into a new virion in the ERGIC. 6) New virion is released by exocytosis. ^79^^–^^82^ Abbreviations. ACE-2: angiotensin-converting enzyme-2; ER: endoplasmic reticulum; ERGIC: endoplasmic reticulum Golgi intermediate compartment; ORF1a: open reading frames 1a; ORF1ab: open reading frames 1ab; pp1a: polyprotein 1a; pp1ab: polyprotein 1ab; RNA: Ribonucleic Acid; SARS-CoV-2: severe acute respiratory syndrome-coronavirus 2; T2DM: type 2 diabetes mellitus; TMPRSS2: Transmembrane protease, serine 2.

Without a doubt, the current pandemic caused by severe acute respiratory syndrome-coronavirus 2 (SARS-CoV-2) represents one of the greatest public health challenges, which has led to extensive worldwide research efforts to identify individuals at greatest risk of developing critical illness. ^1^^–^^3^ The clinical manifestations of the disease caused by SARS-CoV-2, known as coronavirus disease 2019 (COVID-19), are highly variable and range from asymptomatic forms, moderate manifestations and even severe complications, such as: pneumonia, respiratory failure, septic shock, multiple organ dysfunction and death. ^4^^,^^5^ Unfortunately, the molecular mechanisms involved with COVID-19 severity seem to be particularly complex, due to important immunopathological changes induced by SARS-CoV-2; ^6^^–^^9^ as well as metabolic conditions (e.g. obesity, diabetes, hypertension, heart diseases, among others) that underlie the clinical presentation in these patients. ^10^^–^^14^ Regarding latter, a growing body of evidence has suggested that these comorbidities contribute significantly to increased COVID-19 severity and fatal outcomes. ^13^^,^^15^^–^^18^ For instance, several studies have reported that obesity is a comorbidity that increases the risk of complications in SARS-CoV-2 infection, for the following reasons: *(a)* it has been suggested that the ACE-2 receptor expression (target of SARS-CoV-2) is higher in adipose tissue than in the lung parenchyma, which makes adipose tissue an important viral reservoir (see Figure 1); ^19^^,^^20^ *(b)* the Renin-Angiotensin-Aldosterone System ( *RAAS*), a hormonal cascade which regulates blood pressure and is generally overactive in obese patients, has been linked to SARS-CoV-2 cellular infection as well as myocardial and lung injury; ^21^^,^^22^ and *(c)* it is well known that obesity is related to an increase in circulating levels of many adipokines and pro-inflammatory mediators released by adipocytes. ^10^ Therefore, obesity-induced adipose tissue inflammation generates important metabolic abnormalities and disproportionate effects on the immune system, which are relevant pathophysiological aspects in COVID-19 severity. ^2^^,^^23^^,^^24^ On the other hand, in comorbidities like hypertension and type 2 diabetes mellitus (T2DM), besides the previously mentioned points, severe metabolic dysfunctions and several coagulation system alterations take place. ^25^^–^^27^ For example: *(a)* it has been reported that the endothelial dysfunction in obese patients with hypertension promotes the development of a hypercoagulable pro-thrombotic state (by exposure of tissue factor and other pathways), which contributes markedly to life-threatening complications of COVID-19, such as venous thromboembolic disease, systemic vasculitis, endothelial cell apoptosis and multiple organ involvement; ^27^^,^^28^ and *(b)* it is also clear that insulin resistance contributes substantially to the more severe phenotype associated with obesity and T2DM in COVID-19. ^29^^,^^30^ In fact, it has been suggested that the SARS-CoV-2 infection could cause disturbances in glucose metabolism, therefore the acute hyperinflammatory state itself could worsen insulin resistance in these patients. ^10^^,^^30^ In this context, these important pathophysiological alterations in COVID-19 patients have led to an urgent necessity in identifying clinical laboratory predictors, which could provide relevant information in determination of prognosis, patient follow-up, and therapeutic monitoring, as well as differentiate severe patients from the mild/moderate form of COVID-19. ^31^^,^^32^ For instance, biomarkers of an overactive innate immune system, such as markedly elevated neutrophil count, IL-6, C-reactive protein and serum ferritin, could help recognize a potential severe SARS-CoV-2 infection during triage, while biomarkers of organ failure could be helpful in monitoring evolution of COVID-19 patients admitted to the intensive care unit (ICU). ^33^^,^^34^ Thus, both the etiological diagnosis of SARS-CoV-2 and the classification of these patients are the most obvious scenarios in the current health crisis, in which the clinical laboratory plays a fundamental role. ^31^^,^^32^^,^^35^ Because of all the above described, the early identification of comorbid conditions and laboratory predictors associated with the SARS-CoV-2 infection severity, as well as the rapid application of measures to control this infection are currently the main strategies to prevent and reduce the risk of the virus spreading. For this reason, the present study aimed to identify the comorbidities and laboratory parameters among COVID-19 patients admitted to the ICU, comparing those patients who required invasive mechanical ventilation (IMV) to those who did not require IMV, in order to determine the clinical characteristics associated with the COVID-19 severity.

### 1.1 PICOT question

What are the comorbidities and laboratory parameters associated with SARS-CoV-2 infection severity in patients from the southeast of Mexico?

## 2. Methods

### 2.1 Study design

This research used a cross-sectional design.

### 2.2 Participants

The present study enrolled 152 patients diagnosed with COVID-19, who were admitted to the ICU of the General Hospital “Dr. Desiderio G. Rosado Carbajal" from April 1 ^st^ to July 31 ^st^, 2021. For the confirmatory diagnosis of this disease, nasopharyngeal and throat swab specimens were collected upon admission, which were subsequently analyzed by real-time polymerase chain reaction (qPCR) for SARS-CoV-2 RNA detection. Case definitions for these patients were in accordance with the interim guidance of the World Health Organization (WHO), which includes: fever, cough, dyspnea, respiratory frequency ≥ 30/min, SpO2 ≤93%, PaO2/FiO2 ratio <300 and lung infiltrates >50%, as severe clinical manifestations of COVID-19. ^36^ It is necessary to highlight that this is one of the most important hospitals in the southeast of Mexico and has been designated by the Federal Secretary of Health for the hospitalization of COVID-19 patients since February 2020. Identification of these patients was achieved by reviewing and analyzing admission records and clinical histories from all available electronic medical records. Therefore, patients with clinical data of pneumonia, but with negative SARS-CoV-2 test results were excluded from this study. Additionally, since it has been documented that both phenotypic and genotypic characteristics could contribute substantially to the development of specific comorbidities, ^37^^–^^39^ COVID-19 patients from other ethnicities (e.g. Asian, African and Caucasian individuals) were also excluded from the present study.

### 2.3 Ethics and consent statement

This cross-sectional study was conducted according to the guidelines laid down in the Declaration of Helsinki ^40^ and all procedures involving research study participants were requested and verbally approved by the Ethics Commission of the hospital on March 16 ^th^, 2021. Written informed consent was waived by the Ethics Commission of the designated hospital due to the rapid onset of this public health emergency. Verbal consent of the patients was witnessed by a medical professional assigned to the hospital (D.C.E.) and formally documented in the medical record. However, regarding patients who were unable to provide this consent due to their severe clinical condition, a patient's family member provided it, which was then confirmed once the patient was found lucid.

### 2.4 Clinical data and sample collection

Concerning this point, the data collected at the time of admission was the following. 1) Demographic data, including age and gender. 2) Comorbidities, such as T2DM, hypertension, dyslipidemia, chronic kidney disease, heart disease, chronic obstructive pulmonary disease, obesity and malignancy, which were chosen and determined according to the self-administered comorbidity questionnaire (SCQ), ^41^ an instrument that asks about the presence, treatment and functional limitations of 12 common comorbidities and three additional non-specified medical problems. ^41^ 3) Clinical symptoms, which included: fever, cough, sore throat, nasal congestion, breathing difficulties, headache, myalgia, diarrhea, vomiting and nausea. These were measured according to the medical symptom questionnaire (MSQ) that identifies several symptoms which help to find the underlying causes of illness, by using 15 categories in which the patients rate a particular symptom from 0 (never experienced) to 4 (frequently experienced and severe) (available from Lake Travis Integrative Medicine). Here it is important to note that in those cases where the patient was unable to provide the information described above (i.e., comorbidities and clinical symptoms) due to their severe clinical condition (including confused and unconscious states), a patient's family member provided it. 4) Vital signs, such as: temperature, spO2, respiratory and heart rate, which were measured using a monitoring equipment and chosen according to the early warning score (EWS), ^42^ a physiological scoring system based on the individual values of multiple vital signs to quickly evaluate the level of clinical deterioration, in both emergency and general care conditions. ^42^ Approximately 15 minutes after admission, the blood samples were collected and sent to the laboratory to be analyzed according to standard methods using equipment like AcT5diff hematology analyzer (Beckman Coulter), Synchron LX-20 chemistry analyzer (Beckman Coulter) and Lumipulse G600II analyzer (Fujirebio). Briefly, the laboratory tests and how they were measured are mentioned below: complete blood count (CBC) determined by electrical impedance; blood chemistry and liver function test, whose parameters were measured by colorimetry methods; serum electrolytes determined by potentiometry and electrical conductivity method; C-reactive protein (CRP), interleukin 6 (IL-6) and lactate dehydrogenase (LDH) as inflammation-related biomarkers, which were measured by turbidimetric, chemiluminescent and kinetic UV method, respectively. Thus, these laboratory tests were part of the standard of medical care. Finally, all the data mentioned in this section was recorded in an electronic database by two independent researchers (D.C.E. and J.G.C.) and verified by two experienced doctors (G.G.J.A. and L.V.C.J.).

### 2.5 Statistical analysis

With the purpose of understanding the comorbidities and laboratory parameters associated with the SARS-CoV-2 infection severity in patients from the Mexican southeast, the data collected was grouped into two main groups: patients who required invasive mechanical ventilation (IMV) and patients who did not require IMV. The continuous data was described as mean and standard error, while categorical data was described as percentages. The nonparametric Mann–Whitney U test for continuous data and *χ ^2^* test for categorical data were used to compare variables between both groups. On the other hand, in order to evaluate the laboratory parameters in predicting the COVID-19 severity, the Receiver Operating Characteristic (ROC) curves were plotted corresponding to the variables found to show significance, with the corresponding areas under the curve (AUC), sensitivity, specificity, 95% confidence intervals (95%CI), as well as the optimal cutoff, which was defined as the value maximizing the Youden index. A *p* value <0.05 was considered statistically significant. All statistical analyses were performed using SPSS Statistics version 23.0 software, and figures created with SPSS and CorelDRAW graphics suite 2020, or Inkscape 0.92 could also be used as an alternative. Lastly, it is important to mention that a large number of patients who were included in this research (n=127) had incomplete data in their medical records concerning socioeconomic status, sanitary conditions, physical activity, nutritional habits, household income and access to healthcare services, so it was decided not to capture this information in the database, in order to reduce biases in the interpretation of the results.

## 3. Results

### 3.1 Demographic and comorbidity data

As shown in Table 1, 152 COVID-19 patients (men n=92; mean age=59.62) were admitted to the ICU, of whom 66 required IMV (men n=43; mean age=59.80) and 86 did not require IMV (men n=49; mean age=59.48). Regarding comorbidities observed, the ICU-admitted patients who required IMV showed a higher prevalence of T2DM, hypertension and obesity compared to those who did not require IMV ( *p<0.05*). In contrast, here it should be emphasized that while dyslipidemia was a prevalent condition among the COVID-19 patients admitted to the ICU (n=67), this did not show significant differences when both patient groups were compared. Finally, the least prevalent comorbidities in the whole sample were as follows: chronic kidney disease (n=8), heart disease (n=5), chronic obstructive pulmonary disease (n=12) and malignancy (n=1), in which no significant differences were found either.

**Table 1.** Clinical characteristics of ICU-admitted patients infected with SARS-CoV-2.

	All patients (n=152)	Invasive mechanical ventilation		p-value
		No (n=86)	Yes (n=66)	
**Demographic data**				
Mean age, years ±Standard error	59.62±0.93	59.48±1.17	59.80±1.52	0.92
Men, n (%)	92.0 (60.52)	49.0 (56.97)	43.0 (65.15)	0.30
**Comorbidities**				
T2DM, n (%)	92.0 (60.52)	36.0 (41.86)	56.0 (84.84)	<0.05
Hypertension, n (%)	58.0 (38.15)	16.0 (18.60)	42.0 (63.63)	<0.05
Dyslipidemia n (%)	67.0(44.07)	35.0 (40.69)	32.0(48.48)	0.33
CKD, n (%)	8.0 (5.26)	3.0 (3.48)	5.0 (7.57)	0.26
Heart disease, n (%)	5.0 (3.28)	4.0 (4.65)	1.0(1.51)	0.28
COPD, n (%)	12.0 (7.89)	5.0(5.81)	7.0(10.60)	0.27
Obesity, n (%)	101.0(66.44)	38.0(44.18)	63.0 (95.45)	<0.05
Malignancy, n (%)	1.0(0.65)	1.0 (1.16)	0.0 (0.00)	0.37
**Clinical symptoms**				
Fever, n (%)	134.0 (88.15)	74.0 (86.04)	60.0 (90.90)	0.35
Cough, n (%)	127.0 (83.55)	69.0 (80.23)	58.0 (87.87)	0.20
Sore throat, n (%)	93.0 (60.78)	56.0 (65.11)	37.0 (56.06)	0.25
Nasal congestion, n (%)	91.0 (59.86)	49.0 (56.97)	42.0 (63.63)	0.40
Breathing difficulty, n (%)	120.0 (78.94)	57.0 (66.27)	63.0 (95.45)	<0.05
Headache, n (%)	79.0 (51.97)	47.0(54.65)	32.0 (48.48)	0.45
Myalgia, n (%)	44.0(28.94)	25.0(20.06)	19.0 (28.78)	0.97
Diarrhea, n (%)	8.0 (5.26)	5.0 (5.81)	3.0 (4.54)	0.72
Vomit, n (%)	6.0 (3.94)	4.0 (4.65)	2.0(3.03)	0.61
Nausea, n (%)	11.0 (7.23)	7.0 (8.13)	4.0(6.06)	0.62
**Vital signs, mean ± Standard Error**				
Temperature, °C	38.93±0.07	38.94±0.10	38.92±0.10	0.91
spO _2_, % (Normal range: 95.0-100.0%)	80.75±5.82	84.11±0.41	76.36±0.62	0.02
Respiratory rate, brpm (Normal range: 15-20 brpm)	28.59±0.13	27.96±0.11	29.40±0.22	0.04
Heart rate, bpm (Normal range: 60-100 bpm)	87.46±0.32	86.89±0.36	88.19±0.56	0.66

Abbreviations: brpm: breaths per minute **;** bpm: beats per minute; COPD: Chronic obstructive pulmonary disease; CKD: Chronic kidney disease; spO _2_: blood oxygen saturation; SARS-CoV-2: severe acute respiratory syndrome-coronavirus 2; T2DM: type 2 diabetes mellitus.

### 3.2 Clinical symptoms and vital signs

Regarding the clinical symptoms, we observed that the COVID-19 patients admitted to the ICU showed common symptoms of acute respiratory infection (see Table 1), such as: fever (88.15%), cough (83.55%), sore throat (60.78%), nasal congestion (59.86%), breathing difficulty (78.94%), headache (51.97%) and myalgia (28.94%); however, of all these symptoms, only breathing difficulty was significantly higher in those patients who required IMV (p< 0.05). On the other hand, digestive symptoms such as: diarrhea, vomit and nausea were less frequent (5.26%, 3.94% and 7.23%, respectively), and no significant differences were observed either. According to vital signs, as was expected, a lower oxygen saturation and a higher respiratory rate were observed among patients who required IMV than those who did not require IMV (p<0.05). Finally, no significant differences in temperature and heart rate were observed between both patient groups.

### 3.3 Laboratory parameters

As observed in Table 2, several hematological and biochemical alterations were found among the ICU-admitted patients. For example, regarding the complete blood count, (CBC) an accentuated lymphopenia (11.76%) and a high neutrophil count (81.22%) were observed in these patients; however, when both patient groups were compared, no significant differences were found in these hematological parameters. Likewise, the results of blood chemistry showed elevated levels of glucose, cholesterol and triglycerides among these patients (169.87mg/dL, 202.05 mg/dL and 155.22 mg/dL, respectively); nevertheless, only glucose levels were significantly higher in those patients who required IMV than those who did not require IMV (p<0.05). With regard to liver function test performed among the ICU-admitted patients, only a slight increase in transaminases levels (ALT=51.44 U/L; AST=68.07 U/L) was observed; however, no significant differences were found in these enzymatic parameters either. Concerning the inflammation related-biomarkers, a marked elevation in IL-6, CRP and LDH levels was observed among the ICU-admitted patients (183.59 pg/mL, 267.79 mg/L and 481.32 U/L, respectively), which were significantly higher in those patients who required IMV than those who did not require IMV (p<0.05). Finally, no significant changes in electrolyte levels were observed among the COVID-19 patients admitted to the ICU.

**Table 2.** Laboratory parameters of ICU-admitted patients infected with SARS-CoV-2.

	Normal range	All patients (n=152)	Invasive mechanical ventilation		p-value *
			No (n=86)	Yes (n=66)	
**Complete blood count, mean ± Standard Error**					
WBC, ×10 ^3^/μL	4.50-11.0	10.64±0.36	10.32±0.50	11.06±0.53	0.90
Neutrophils, ×10 ^3^/μL	1.80-7.0	8.97±0.36	8.75±0.52	9.27±0.47	0.18
Neutrophils, %	50.0-70.0	81.22±1.02	79.93±1.67	82.91±0.89	0.60
Lymphocyte, ×10 ^3^/μL	1.0-4.80	1.0±0.03	0.95±0.04	1.06±0.05	0.91
Lymphocyte, %	20.0-45.0	11.76±0.73	12.76±1.20	10.45±0.62	0.39
Eosinophils, ×10 ^3^/μL	0.10-0.45	0.02±0.01	0.03±0.01	0.01±0.01	0.06
Eosinophils, %	1.0-4.0	0.45±0.11	0.58±0.18	0.28±0.12	0.23
Hemoglobin, g/dL	13.0-16.50	13.40±0.15	13.25±0.20	13.59±0.23	0.15
Platelets, ×10 ^3^/μL	140.0-400.0	292.96±5.15	292.88±6.01	293.07±8.97	0.08
**Blood chemistry, mean ± Standard Error**					
Glucose, mg/dL	70.0-110.0	169.87±6.41	144.77±8.65	202.57±7.94	<0.001
Urea, mg/dL	15.0-40.0	38.87±1.44	41.04±2.51	36.04±0.35	0.97
Creatinine, mg/dL	0.60-1.20	0.96±0.08	1.10±0.14	0.78±0.02	0.72
uric acid, mg/dL	3.50-8.50	5.27±0.08	5.15±0.10	5.44±0.14	0.07
Total cholesterol, mg/dL	70-200.0	202.05±2.69	200.87±3.71	203.59±3.91	0.57
Triglycerides, mg/dL	65-165	155.22±5.53	151.59±6.74	159.95±9.27	0.65
**Liver function test, mean ± Standard Error**					
Albumin, g/dL	3.50-5.0	3.64±0.03	3.67±0.03	3.61±0.06	0.06
ALT, U/L	0.0-50.0	51.44±4.27	64.61±7.01	34.28±2.43	0.06
AST, U/L	17.0-59.0	68.07±5.02	74.97±8.60	59.07±2.60	0.19
**Serum electrolytes, mean ± Standard Error**					
Sodium, mmol/L	137.0-145.0	137.84±0.50	138.66±0.81	136.78±0.46	0.66
Potassium, mmol/L	3.50-5.10	4.61±0.06	4.59±0.07	4.64±0.11	0.74
Chlorine, mmol/L	98.0-107.0	101.67±0.50	102.19±0.79	100.98±0.54	0.86
Total calcium, mg/dL	8.40-10.20	8.52±0.02	8.48±0.03	8.56±0.05	0.11
Phosphorus, mg/dL	2.50-4.50	3.66±0.05	3.55±0.04	3.81±0.10	0.07
Magnesium, mg/dL	1.60-2.30	2.03±0.03	1.99±0.04	2.08±0.06	0.54
**Inflammation-related biomarkers, mean ± Standard Error**					
IL-6, pg/mL	0.0-3.40	183.59±5.08	166.70±6.39	205.60±7.46	<0.001
CRP, mg/L	0.0-10.0	267.79±5.33	254.78±7.05	284.74±7.73	0.001
LDH, U/L	91.0-180.0	481.32±11.54	453.79±13.22	517.21±19.49	0.020

Abbreviations: ALT: alanine transaminase; AST: aspartate transaminase; CRP: C-reactive protein; IL-6: interleukin-6; LDH: lactate dehydrogenase; WBC: White blood cell.

* Determined by Mann-Whitney U test for independent samples.

### 3.4 ROC curve analysis

**Figure. 2.**
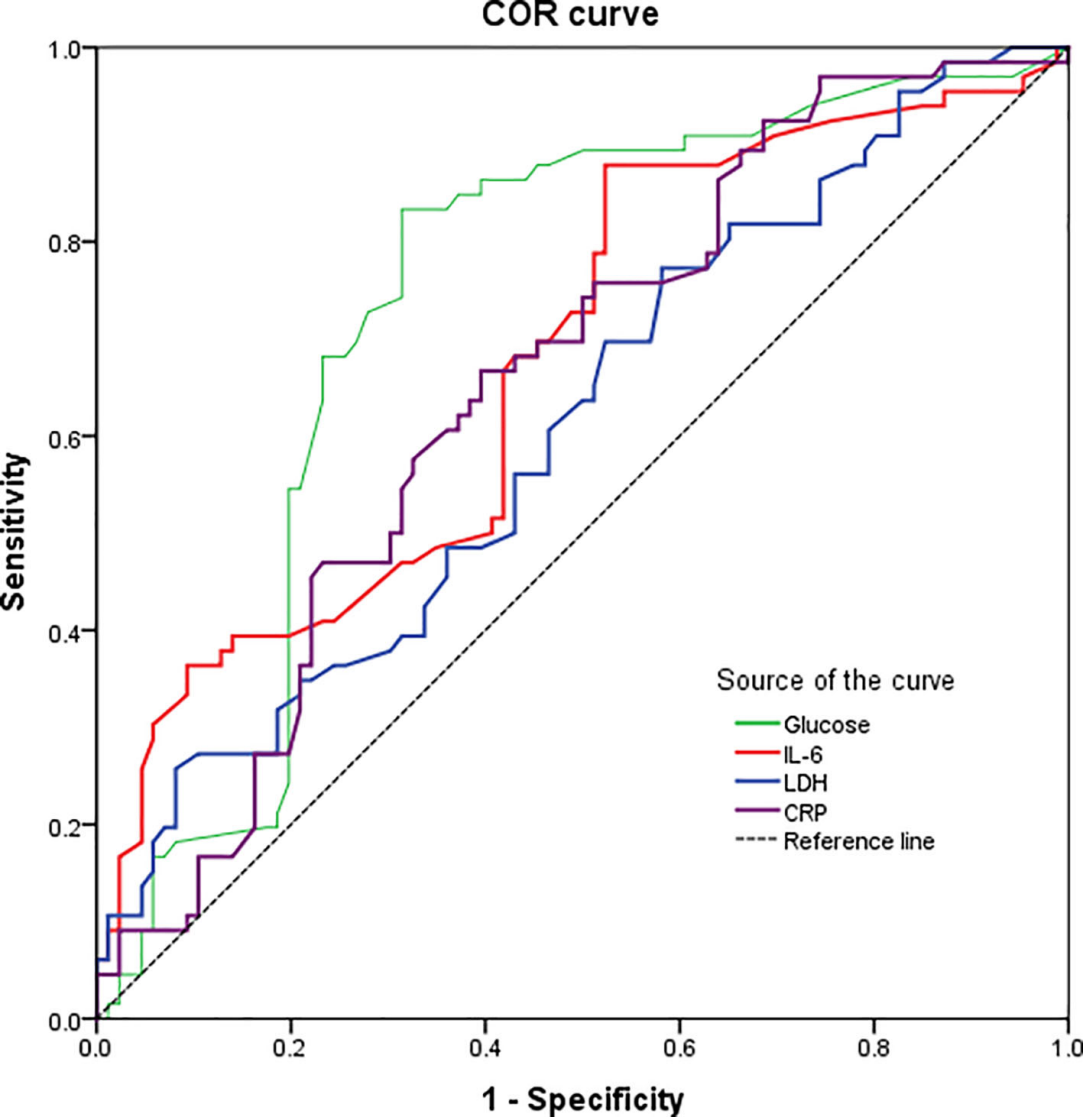
Receiver operating characteristics (ROC) curve of significant laboratory parameters showing the predictive power of glucose, IL-6, LDH and CRP in the COVID-19 severity. Abbreviations: CRP: C-reactive protein; IL-6: interleukin-6; LDH: lactate dehydrogenase.

We performed ROC curves on the above laboratory parameters with significant differences to assess their value predictive in the COVID-19 severity ( Figure 2). For instance, the IL-6 was the most specific predictor (specificity 95.3%) with a high sensitivity (97.0 %) for COVID-19 severity, based on a cut-off of 84.1 pg/mL and an area under the curve (AUC) of 0.675 (95% CI: 0.588-0.761). Similarly, the CRP levels showed a 98.5% sensitivity and 94.2% specificity for predicting severe COVID-19, based on an AUC of 0.651 (95% CI: 0.564-0.738) and a cut-off of 154.6 mg/L. Likewise, LDH levels showed a 95.5 % sensitivity and 84.9 % specificity for predicting severe COVID-19, based on an AUC of 0.610 (95% CI: 0.520-0.700) and a cut-off of 325.0 U/L. In contrast, the glucose levels showed a high sensitivity (86.4%) but a very poor specificity (40.7%) for the COVID-19 severity, based on an AUC of 0.736 (95% CI: 0.653-0.818) and a cut-off of 116.0 mg/dL. All the above data are described in Table 3.

**Table 3.** ROC curve analysis of clinical laboratory data.

Laboratory parameters	Cutoff value *	Specificity	Sensitivity	AUC	95% CI	p-value
Glucose	116.0 mg/dL	40.7%	86.4%	0.736	0.653-0.818	<0.001
IL-6	84.1 pg/mL	95.3%	97.0 %	0.675	0.588-0.761	<0.001
LDH	325.0 U/L	84.9%	95.5%	0.610	0.520-0.700	0.020
CRP	154.6 mg/L	94.2%	98.5 %	0.651	0.564-0.738	0.001

Abbreviations: AUC: Area under the curve; CRP: C-reactive protein; IL-6: interleukin-6; LDH: lactate dehydrogenase.

* Defined as the value maximizing the Youden index.

## 4. Discussion

In this paper, the comorbidities and laboratory parameters associated with SARS-CoV-2 infection severity in patients from the Mexican southeast were analyzed. Regarding the comorbidities found in our study, a higher prevalence of obesity, T2DM and hypertension in those patients who required IMV was observed (p<0.05). These findings attract a lot of attention, since unfortunately Mexico is among the highest places in terms of prevalence of these comorbidities, ^43^^,^^44^ which could partially explain the high hospital mortality rate related to COVID-19 in this country; in fact, according to data compiled by John Hopkins University, until October 2021, more than 284,381 deaths had been registered in Mexico. In this context, several studies support our findings, in which it has been documented that COVID-19 patients suffering from these metabolic disorders increase the need for critical care and particularly for IMV requirement. ^45^^–^^50^ For instance, Simonnet *et al*. ^47^ and Costa Monteiro *et al*. ^48^ reported an elevated prevalence of obesity and T2DM among ICU-admitted patients infected with SARS-CoV-2. These studies indicated that these comorbidities could serve as clinical predictors for risk stratification models. Likewise, these studies concluded that early measurement of anthropometric and metabolic parameters in these patients could be crucial to avoid unfavorable clinical outcomes. ^47^^,^^48^ Similarly, Busetto *et al.*, ^45^ Cummings *et al.* ^46^ and Borobia *et al*. ^50^ evaluated the clinical course of critically ill patients with COVID-19. In summary, these studies reported that COVID-19 patients with obesity, T2DM and hypertension showed a higher risk of more severe clinical symptoms and extrapulmonary organ dysfunction during SARS-CoV-2 infection, thus requiring a more frequent need for ICU admission and IMV. ^45^^,^^46^ Likewise, Petrilli *et al*. ^49^ conducted a study including more than 4000 COVID-19 cases, in which obesity was the strongest predictor of critical illness, substantially higher than pulmonary or cardiovascular diseases. ^49^ In this regard, precise pathophysiological mechanisms related to a higher prevalence of these comorbidities among COVID-19 patients requiring IMV are not completely understood. However, recently several studies have reported that these metabolic disorders are multifactorial conditions that are closely associated with severe respiratory dysfunctions as well as impaired molecular mechanisms that could worsen the course of SARS-CoV-2 infection. ^51^^,^^52^ For example, *(a)* it is clear that obesity causes mechanical compression of the diaphragm, thoracic cavity and lungs, which could lead to a restrictive pulmonary damage and consequently to an impaired respiratory ventilation. ^53^^,^^54^ Moreover, several studies have indicated that an excessive adipose tissue amount in the abdominal area reduces the strength of the respiratory muscles, decreases the total compliance of the respiratory system and increases pulmonary resistance. ^54^^–^^56^ *(b)* It has been reported that obese patients with hypertension are more likely to develop severe respiratory diseases, such as: asthma, ^57^ chronic obstructive pulmonary disease, ^58^^,^^59^ obesity hypoventilation syndrome, ^60^^,^^61^ pulmonary hypertension and obstructive sleep apnea, ^62^^,^^63^ which predisposes them to low levels of blood oxygenation and evidently to fatal respiratory outcomes in severe SARS-CoV-2 infection. ^64^^,^^65^ *(c)* T2DM could negatively impact clinical outcomes in COVID-19 patients admitted to the ICU, since it has been documented that hyperglycemia in diabetic patients could increase SARS-CoV-2 replication, at the same time aerobic glycolysis could facilitate SARS-CoV-2 replication via synthesis of mitochondrial reactive oxygen species and activation of hypoxia-inducible factor 1α. ^66^^,^^67^ Thus, alterations in glucose metabolism could also have influenced a greater need for IMV as well in the poor prognosis in these patients. ^66^^,^^67^ On the other hand, several hypotheses have emerged suggesting that SARS-CoV-2 could also be a key contributor in the worsening of metabolic status in comorbid patients requiring IMV, for example: *(a)* acute inflammatory state induced by SARS-COV-2 could alter the lipid and glucose metabolism. This hypothesis is supported by the fact that pro-inflammatory cytokines (e.g. IL-1β, IL-6 and TNF-α) modulate the metabolism of these biomolecules; hence, dyslipidemia and hyperglycemia observed in the ICU-admitted patients could also be due to an inadequate cellular secretion of cytokines and/or an inappropriate immune response induced by SARS-CoV-2. ^68^^,^^69^ *(b)* Oxidative stress promoted by SARS-COV-2 infection could exacerbate dyslipidemia in COVID-19 patients with underlying metabolic disorders. This argument arises from the fact that most viral infections manipulate antioxidant systems in several chronic conditions, leading to abnormalities in cellular metabolism. ^70^^–^^72^ *(c)* SARS-CoV-2 could directly affect liver function and thus alter the lipid biosynthesis. This hypothesis could partially explain the biochemical changes found in our study, in which a slight increase in serum aspartate transaminase (AST) and alanine transaminase (ALT) levels was observed. However, these slight changes likely do not contribute significantly to the increased levels of cholesterol and triglycerides in the COVID-19 patients admitted to the ICU. Finally, in our study the elevated levels of CRP, IL-6 and LDH were the most specific and statistically significant parameters in both groups of patients, which suggests that these molecules could play a key role during the progression and the prognosis of fatal outcomes in COVID-19 patients who required IMV. ^5^^,^^73^^,^^74^ In fact, these biomarkers have previously been associated with the severity and mortality of COVID-19 in most cases defined by the Chinese National Health Commission. ^75^^–^^77^ Moreover, recent publications have provided additional information that strengthen the role of CRP, IL-6 and LDH as predictive markers of SARS-CoV-2 infection severity, especially in critically ill patients. ^75^^,^^77^^,^^78^

A strength of this research is that it provides scientific evidence indicating that comorbidities such as obesity, T2DM, and hypertension, as well as elevated levels of glucose, IL-6, LDH and CRP are associated with the COVID-19 severity among ICU-admitted patients. Moreover, the clinical and laboratory data was collected from one of the most important hospitals in the Mexican southeast, which concentrates a large part of COVID-19 patients in that region. Finally, our study has some limitations inherent to methodological design that could affect the interpretation of results. First, since our study included only COVID-19 patients from the southeast of Mexico, one needs to be careful to extrapolate our findings to those who reside in other geographical areas of the country and the world. Second, only the basal clinical findings were included in this research, while the clinical changes induced by disease progression, pharmacological treatment and invasive mechanical ventilation were not addressed in this paper, which could lead to important biases of clinical observation and interpretation in these patients. Third, the small number of patients included in this study limit the ability to determine causal inferences linked to the COVID-19 severity. Therefore, randomized clinical trials and observational studies (with a larger number of patients) addressing the factors underlying the severe conditions of COVID-19 in patients with obesity, hypertension, T2DM and/or metabolic dysfunction could contribute to determine the causes associated with the clinical progression and severity of this disease. Fourth, the retrospective design of the present study is also an important limitation, since a large number of cases included in this study had incomplete data in their medical records (e.g. information on physical activity, socioeconomic status, nutritional habits, hygienic conditions, access to healthcare services as well as household income); thus, it was not possible to adjust the risks associated with the COVID-19 severity in these patients. Besides, it is likely that the categorical stratification used in our study was not the most appropriate method, since a validated severity scale for COVID-19 patients admitted to ICU was not used; hence, the results presented in this research should be viewed with caution.

## 5. Conclusion

In conclusion, the results of this retrospective case study performed in a Mexican population indicates that metabolic disorders such as: obesity, T2DM, and hypertension, as well as elevated levels glucose, IL-6, LDH and CRP are associated with the SARS-CoV-2 infection severity. Therefore, patients suffering from these conditions should take additional measures to avoid COVID-19 infection by enforcing prevention during the current pandemic. Likewise, public health policies and social support services should focus on disadvantaged communities with high rates of obesity, T2DM, hypertension and nutritional disorders to promote healthy lifestyle choices and preventive strategies that help minimize the risks and health consequences of these diseases, including COVID-19 complications. Moreover, as further waves of the pandemic and new variants of faster spread than early forms of SARS-CoV-2 are expected, improvement of guidelines for individuals with these comorbidities is strongly recommended. Finally, our characterization provides a quick clinical guidance to stratify high susceptibility patients in SARS- CoV-2 infections.

## Data availability

### Underlying data

Harvard Dataverse: Comorbidities and laboratory parameters associated with SARS-CoV-2 infection severity in patients from the southeast of Mexico: A cross-sectional study. https://doi.org/10.7910/DVN/DFALL6 ^83^

This project contains the following files:

- COVID19_Database (v1).tab (Data on clinical features and laboratory parameters of COVID-19 patients).
- Data key.docx (Data key for variables and abbreviations in the tab file)

Data are available under the terms of the Creative Commons Zero “No rights reserved” data waiver (CC0 1.0 Public domain dedication).
