# Supplementary material for: Comorbidities and laboratory parameters associated with SARS-CoV-2 infection severity in patients from the southeast of Mexico: a cross-sectional study
Source: F1000Res. 2022 Apr 27;11:10. Originally published 2022 Jan 6. [Version 2] doi: 10.12688/f1000research.74023.2 (PMC9005987; doi:10.12688/f1000research.74023.2)
Supplement: Peer review history

# Peer review: Reviewer response for version 1

**DOI:** 10.5256/f1000research.77730.r125705

**Published:** 2022-04-12

**Authors:** Perminder Gulani

**Affiliations:** 1 Department of Medicine, Jacobi Medical Center, Bronx, NY, USA

I enjoyed reading this article evaluating comorbidities and laboratory parameters associated with Severe COVID-19 disease.

 This study re-enforces many findings that are already well known in COVID-19. The study enrolled 152 COVID-19 patients admitted to ICU. RT-PCR was used to confirm the diagnosis of COVID. Patients requiring Invasive Mechanical Ventilation were classified as Severe COVID-19 cases. The comparison was made between patients who required invasive mechanical ventilation with those who did not. 

**Limitations of the study:**

-  A small sample size of 152 patients. Many studies looking at the same question have been done on much larger populations. 
- I agree with Invasive Mechanical Ventilation as the criteria for severity--however, there is some subjectivity in deciding who will be intubated. Therefore, this is a second but less critical limitation. 
- The question is not novel--many findings are well known to us. It is well known that patients with obesity, type-2 diabetes, and those with higher CRP and IL-6 have more severe diseases. 

Is the work clearly and accurately presented and does it cite the current literature?

Yes

If applicable, is the statistical analysis and its interpretation appropriate?

Yes

Are all the source data underlying the results available to ensure full reproducibility?

Yes

Is the study design appropriate and is the work technically sound?

Yes

Are the conclusions drawn adequately supported by the results?

Yes

Are sufficient details of methods and analysis provided to allow replication by others?

Yes

Reviewer Expertise:

COVID-19, Sepsis, Cardiomyopathy.

I confirm that I have read this submission and believe that I have an appropriate level of expertise to confirm that it is of an acceptable scientific standard.

# Response

**Published:** 2022-04-14

**Authors:** JOSE ARNOLD GONZALEZ-GARRIDO

**Affiliations:** UNIVERSIDAD JU´ÁREZ AUTÓNOMA DE TABASCO, Mexico

**Reviewer 1 wrote:**

  

1. “No details of the analysis procedures of blood laboratory parameters were included, making the study replicable. Authors may add the detailed methodology at least for glucose, Interleukin 6, lactate dehydrogenase, and C-reactive protein or add the general method of the overall analysis.”

**Reply:** *Thank you very much for this important observation. Consequently, in this version of the manuscript the analyzers and methods used to determine the laboratory parameters have been mentioned at the end of the section of clinical data and sample collection.*

1. “The blood parameters were detected on the blood samples collected after 15min of admission of the patients, which makes it difficult to study to connect with the severity of COVID-19. If there is an opportunity to add the follow-up blood sample parameters like after 15 days of admission, the comparison with severity will be recognizable.”

**Reply:** *We appreciate your valuable comment. However, due to the nature of this research (cross-sectional study), a follow-up study of these patients was not considered* *(see second limitation at the end of the discussion section).*

**3. **“Some grammatical errors were found (e.g., it has reported that: correct form: it has been…., conclusion section: indicates- correct form: indicate, etc.) that should be corrected.”

**Reply:** *Thank you very much for your kind observation. Consequently, these grammatical errors have been corrected.*

# Peer review: Reviewer response for version 1

**DOI:** 10.5256/f1000research.77730.r119989

**Published:** 2022-02-11

**Authors:** Mohammad Safiqul Islam

**Affiliations:** 1 Department of Pharmacy, Noakhali Science and Technology University, Noakhali, Bangladesh

The manuscript entitled “Comorbidities and laboratory parameters associated with SARS-CoV-2 infection severity in patients from the southeast of Mexico: a cross-sectional study” submitted by Cruz-Cano *et al.* evaluated the connection of comorbidities and laboratory parameters with the severity of SARS-CoV-2 infection. The authors recruited 152 ICU admitted COVID-19 patients, and among them, 66 required invasive mechanical ventilation (IMV), whereas 86 did not require IMV. Among the comorbidities, the authors found a connection of diabetes mellitus (T2DM), hypertension, and obesity with COVID-19 severity, whereas, among the laboratory parameters, glucose, Interleukin 6 (IL-6), lactate dehydrogenase (LDH), and C-reactive protein (CRP) were associated with the severity of COVID-19 considering the requirement of IMV. The manuscript has substantial merit for indexing. However, some points should be clarified from the authors' side.

  

1. No details of the analysis procedures of blood laboratory parameters were included, making the study replicable. Authors may add the detailed methodology at least for glucose, Interleukin 6, lactate dehydrogenase, and C-reactive protein or add the general method of the overall analysis.
2. The blood parameters were detected on the blood samples collected after 15min of admission of the patients, which makes it difficult to study to connect with the severity of COVID-19. If there is an opportunity to add the follow-up blood sample parameters like after 15 days of admission, the comparison with severity will be recognizable.
3. Some grammatical errors were found (e.g., it has reported that: correct form: it has been…., conclusion section: indicates- correct form: indicate, etc.) that should be corrected.

Is the work clearly and accurately presented and does it cite the current literature?

Yes

If applicable, is the statistical analysis and its interpretation appropriate?

Yes

Are all the source data underlying the results available to ensure full reproducibility?

Yes

Is the study design appropriate and is the work technically sound?

Yes

Are the conclusions drawn adequately supported by the results?

Yes

Are sufficient details of methods and analysis provided to allow replication by others?

Partly

Reviewer Expertise:

Pharmacology, Clinical Pharmacy, Pharmacogenomics, cancer genomics

I confirm that I have read this submission and believe that I have an appropriate level of expertise to confirm that it is of an acceptable scientific standard.

# Response

**Published:** 2022-04-14

**Authors:** JOSE ARNOLD GONZALEZ-GARRIDO

**Affiliations:** UNIVERSIDAD JU´ÁREZ AUTÓNOMA DE TABASCO, Mexico

**Reviewer 2 wrote:**

  

- “A small sample size of 152 patients. Many studies looking at the same question have been done on much larger populations.”

**Reply:** *Thank you very much for your kind comment. For this reason, the small sample size has been included in our study as an important limitation (see third limitation at the end of the discussion section).*

- “I agree with Invasive Mechanical Ventilation as the criteria for severity--however, there is some subjectivity in deciding who will be intubated. Therefore, this is a second but less critical limitation.”

**Reply:** *We deeply appreciate your kind comment. Although it is true that in our paper we did not use a validated severity scale (as specified in the fifth and last limitation at the end of the discussion section), the criteria for invasive mechanical ventilation were based on clinical characteristics and laboratory parameters of these patients (e.g. blood oxygen saturation, respiratory rate, IL-6, CRP, etc), which have also been used as severity predictors in similar studies, and recently included in validated scales as severity criteria for COVID-19.*

**Reviewer 2 wrote:**

-  “The question is not novel--many findings are well known to us. It is well known that patients with obesity, type-2 diabetes, and those with higher CRP and IL-6 have more severe diseases.”

**Reply:** *Thank you very much for your kind comment*. *Although this study shows many findings that are well known to the scientific community, we believe that this small research could modestly contribute to the reinforcement of the current evidence.*
